# Invasive and Non-invasive Dynamic Parameters to Predict Fluid Responsiveness After Off-pump Coronary Surgery

**DOI:** 10.5152/TJAR.2021.20411

**Published:** 2022-02-01

**Authors:** Evgeniia V. Fot, Natalia N. Izotova, Aleksei A. Smetkin, Vsevolod V. Kuzkov, Mikhail Y. Kirov

**Affiliations:** Department of Anaesthesiology and Intensive Care Medicine, Northern State Medical University, Arkhangelsk, Russian Federation

**Keywords:** Cardiac surgery, dynamic parameters, fluid challenge test, fluid responsiveness, heart–lung interaction index, plethysmogram variability index

## Abstract

**Objective:**

This study aimed to assess the predictive value of invasive and non-invasive dynamic parameters for evaluation of fluid responsiveness after off-pump coronary artery bypass grafting.

**Methods:**

Thirty-two adult patients after off-pump coronary surgery were enrolled into a single-center pilot prospective observational study. After arrival to the intensive care unit, all patients received standard fluid challenge test to assess fluid responsiveness. The patients with an increase in cardiac index ≥ 15% after the test were defined as fluid responders. We measured pulse pressure variation using 2 monitoring systems (PPV_PiCCO_ and PPV_NK_), stroke volume variation, heart–lung interaction index, and plethysmogram variability index before and after standard fluid challenge test.

**Results:**

After intensive care unit admission, the absolute values of stroke volume variation, PPV_PiCCO_, PPV_NK_, and heart–lung interaction index were significantly higher among fluid responders (*P* < .05). Response to standard fluid challenge test was predicted by dynamic assessment of PPV_PiCCO_ (area under the curve 0.84), PPV_NK_ (area under the curve 0.71), stroke volume variation (area under the curve 0.77), and heart–lung interaction index (area under the curve 0.77) (*P* < .05). The plethysmogram variability index value did not demonstrate any predictive ability regarding fluid responsiveness (area under the curve 0.5, *P*  = .1).

**Conclusions:**

In patients after off-pump coronary surgery, both invasive parameters such as pulse pressure and stroke volume variations and non-invasive parameter such as heart–lung interaction index are able to predict fluid responsiveness. Thus, these dynamic parameters can be used to guide fluid therapy during the early postoperative period after off-pump coronary surgery.

**Main Points**

- Dynamic parameters (PPV_PiCCO__,_ PPV_NK_), stroke volume variation, and heart–lung interaction index (HLI)) can be used for the evaluation of fluid responsiveness after off-pump coronary artery bypass grafting.
- The presence of bradycardia can compromise the interpretation of HLI after off-pump coronary surgery.
- Plethysmogram variability index does not predict the response to fluid therapy after off-pump coronary artery bypass grafting.

## Introduction

Postoperative fluid therapy remains a challenge in different settings, including cardiosurgical interventions. Fluids should be considered as other drugs, especially in patients with a limited cardiac reserve. On average, less than 50% of patients respond to intravenous fluid therapy after cardiac surgery, thus in many of them, the excessive infusion may lead to lung edema, worsening of gas exchange, decrease in myocardial compliance, low cardiac output, and reduced oxygen delivery.^1-2^ Moreover, hypervolemia is also associated with increased number of complications and negative outcomes.^3^ On the other hand, in case of hypovolemia, fluid administration can increase cardiac preload, counteract tissue hypoxia, and maintain organ function.^4^ For this reason, it is important to define which hemodynamic parameters determine a positive or a negative response to fluid therapy. 

Historically, the static markers of cardiac preload, such as central venous pressure (CVP), pulmonary artery occlusion pressure, or heart volumes, have been used to guide the fluid balance in cardiac surgery.^5-7^ However, the static parameters have a limited predictive value in many situations.^4-8^ Therefore, several dynamic tests and parameters have been introduced to clinical practice. In our recent study, we assessed the efficacy of several dynamic tests in cardiosurgical patients,^9^ but the dynamic parameters also require further evaluation in different settings. Most dynamic parameters are based on heart–lung interactions during mechanical ventilation and have a better value to predict fluid responsiveness compared with static parameters.^3,8,10^ Thus, pulse pressure variation (PPV) and stroke volume variation (SVV) can be obtained from the arterial pressure curve. However, these parameters are invasive and can be associated with specific complications.^11-14^ Among non-invasive parameters, both heart–lung interaction index (HLI) and plethysmogram variability index (PVI) are based on plethysmogram changes during respiration phases. The HLI represents respiratory variation of plethysmogram obtained from pulse oximetry and analyzed by the ventilator. The PVI is also a novel algorithm allowing automated and continuous calculation of the respiratory variations in the pulse oximetry waveform amplitude. However, despite several studies of non-invasive dynamic parameters in cardiac surgery,^15-16^ the role of PVI and HLI for fluid management of patients after off-pump coronary surgery is still unsettled.

Thus, the aim of our study was to assess the predictive value of invasive and non-invasive dynamic parameters for the evaluation of fluid responsiveness after off-pump coronary artery bypass grafting (OPCAB).

## Methods

The study was performed in a 900-bed university hospital (City Hospital #1, Arkhangelsk, Russia). Thirty-two adult patients after elective OPCAB were enrolled in a prospective observational study. The study protocol and the informed consent form were approved by the Ethical Committee of Northern State Medical University (Arkhangelsk, Russia). Written informed consent was obtained from every patient. Exclusion criteria were age <18 and >80 years, morbid obesity with body mass index > 40 kg m^−2^, and constant atrial fibrillation.

All patients were intubated using the standard induction ­technique with sodium thiopental (4 mg kg^−1^), fentanyl (2.5-3.0 µg kg^−1^), and pipecuronium bromide (0.1 mg kg^−1^). Anaesthesia was maintained using sevoflurane (0.5-3.0 vol.% at the end of expiration) and fentanyl (2.0-4.0 µg kg^−1^ h^−1^). Depth of anaesthesia was adjusted to maintain bispectral index (BIS) values between 40 and 60 (LifeScope, Nihon Kohden, Japan).

In all cases, preoxygenation with 80% O_2_ was provided during 3-5 minutes before anaesthesia. After tracheal intubation, patients received a protective volume-controlled ventilation (Dräger Primus, Germany) with tidal volume of 6-8 mL kg^−1^ of predicted body weight, flow of 1 L min-1, and positive end-expiratory pressure of 5 cm H_2_O. The value of FiO_2_ was set to at least 50% or higher to achieve intraoperative SpO_2_ above 95%. The respiratory rate was adjusted to maintain end-tidal CO_2_ values within 30-35 mm Hg. Fluid therapy included an infusion of Ringer’s lactate at rates of 6-7 mL kg^−1^ h^−1^ before and during surgery and 2-3 mL kg^−1^ h^−1^ during the first 6 h after operation.

All patients were operated by the same team of surgeons using Acrobat SUV OM-9000S (Guidant, Santa Clara, Calif, USA) device for stabilization of the heart during OPCAB.

After surgery, all patients were transferred to the postoperative cardiac intensive care unit (ICU) and sedated with continuous infusion of propofol (2-4 µg kg^−1^ h^−1^) to maintain BIS values within 60-70. Respiratory support in ICU was provided by a G5 ventilator (Hamilton Medical, Switzerland) using pressure-controlled ventilation mode with parameters of intraoperative ventilation.

We provided invasive hemodynamic monitoring (PiCCO_2_, Pulsion Medical Systems, Germany; Nihon Kohden, MU-671RK, Japan) to all patients. After the initial stabilization of respiratory and hemodynamic parameters, all patients received 7 mL kg^−1^ of crystalloids within 10 minutes (standard fluid challenge test, sFCT).^17^ We performed monitoring of mean arterial pressure (MAP), SVV, and PPV_PiCCO_ using femoral artery (PiCCO_2_). We also assessed PVV_NK_ using radial artery and Nihon Kohden patient monitor. Heart–lung interaction index (Hamilton G-5, Switzerland) and PVI (Masimo, USA) were assessed non-invasively, using finger probes. Both parameters measure the maximal and minimal plethysmographic waveform amplitudes over a given period of time and calculate the percentage difference between the 2.^15-16^ All parameters were measured and recorded before and after sFCT. In addition, we assessed cardiac index (CI), extravascular lung water index (EVLWI), and global end-diastolic volume index (GEDVI) using transpulmonary thermodilution (PiCCO_2_). During the study, we measured arterial blood gases and lactate concentration. The patients with an increase in CI ≥ 15% after sFCT were defined as fluid responders.^18-20^

After the initial measurements, we initiated weaning from mechanical ventilation. The weaning protocol included a gradual decrease of inspiratory support, followed by a spontaneous breathing trial. After passing the 30-min spontaneous breathing trial, all patients were extubated and received oxygen inhalation via facial mask.

We also assessed the preoperative EuroScore II, duration of postoperative mechanical ventilation, length of ICU stay, and fluid balance immediately after OPCAB and on Day 1.

## Statistical Analysis

For data collection and analysis, we used the Statistical Package for Social Sciences (SPSS), version 17.0 software (SPSS Inc.; Chicago, IL, USA). Due to the pilot design of the study, the sample size was limited to 32 patients. All the variables were expressed as median (25th-75th interquartile interval). The groups were compared using Mann–Whitney *U* test. The intragroup comparisons were performed by Friedman and post hoc Wilcoxon tests with Bonferroni correction. Nominal data were compared using χ^2^ test and expressed as patient number. The correlation analysis was performed using rho Spearman. To evaluate the prognostic value of dynamic parameters, we performed receiver operating characteristic (ROC) curve analysis and calculated area under the ROC curve (AUC). The optimal cut-off point for dynamic parameters was determined by maximum value of the Youden Index (maximizing sensitivity and specificity). For post hoc intragroup comparisons, *P* value < .01 was considered as statistically significant. In all other cases, *P* value < .05 was regarded as statistically significant.

## Results

We enrolled 22 males and 10 females in the study. Main demographic and clinical data are presented in Table 1. We observed a significant increase of CI after the sFCT in 44% of patients. Only 2 responders received vasopressor support during the first hour after ICU admission. All patients survived 28 days after surgery.

**Figure 1.**
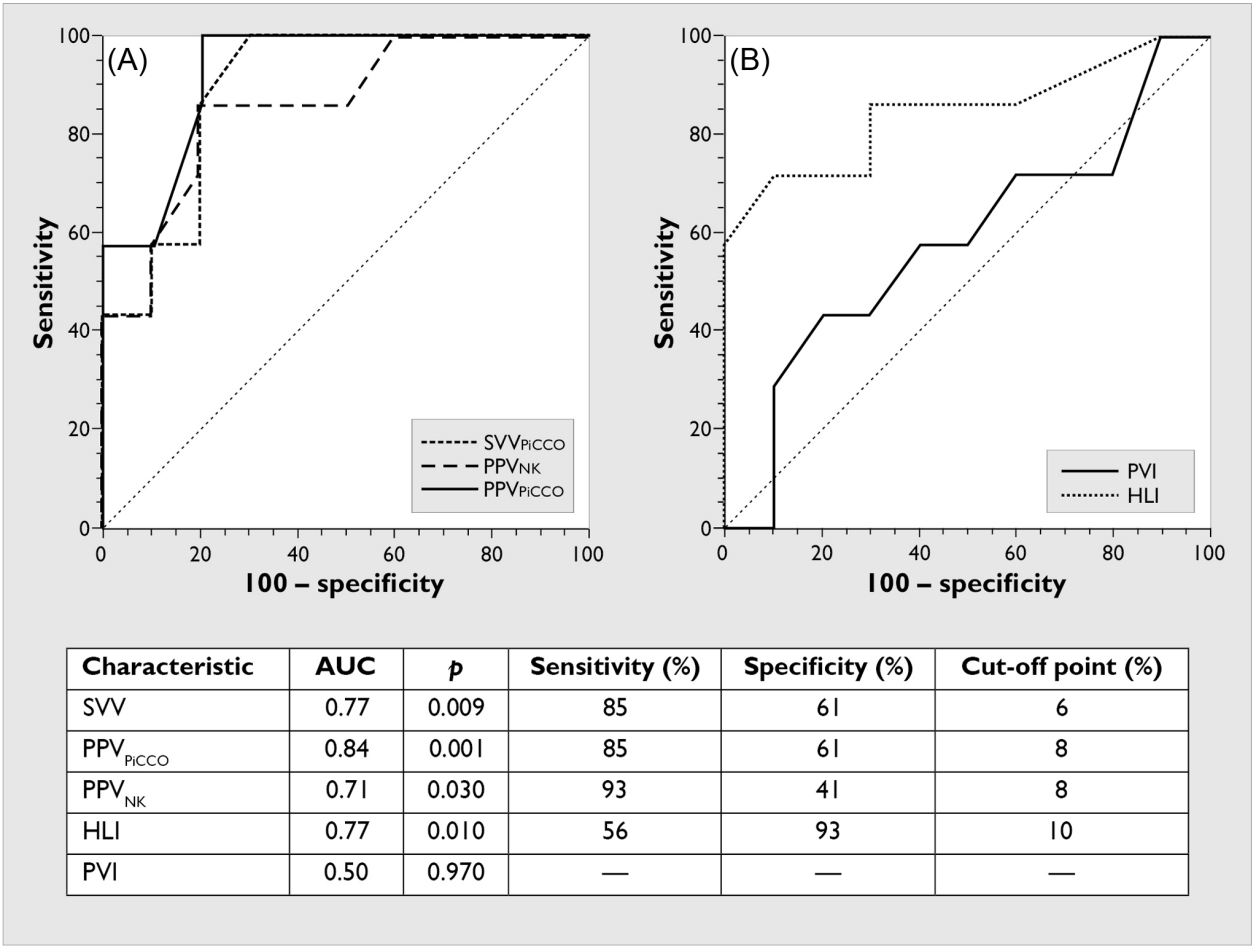
The ROC for invasive (A) and non-invasive (B) dynamic parameters as predictors of fluid responsiveness following the standard fluid load test after off-pump coronary artery bypass grafting. PPV_PiCCO_, pulse pressure variation (PiCCO); PPV_NK_, pulse pressure variation (Nihon Kohden); SVV, stroke volume variation; HLI, heart–lung interaction index; PVI, plethysmogram variability index; AUC, area under the curve; ROC, receiver operating characteristic curve.

At baseline, we observed significantly higher values of SVV, PPV_PiCCO_, PPV_NK_, and HLI in fluid responders (Table 2). There was no baseline difference in PVI and CI between responders and non-responders. Dynamic assessment of these parameters before and after sFCT has also shown an acceptable predictive value (Figure 1), with the best AUC for PPV_PiCCO_. After fluid load, SVV, PPV_PiCCO_, PPV_NK_, HLI, and PVI in the group of responders reduced to normal values without difference from non-responders. However, PVI value before sFCT, as well as dynamic changes in PVI after sFCT, did not demonstrate any predictive ability regarding fluid responsiveness.

We observed difficulties in registration of PVI after admission to the ICU in 3 patients. We also had difficulties in registration of HLI in 7 patients with bradycardia. Interestingly, these patients had a hazard ratio (HR) of 47 (41-51) beats/min, whereas in patients with normal HLI, signal HR was 69 (57–78) beats/min (*P* < .001).

As expected, the fluid load test was accompanied by the increase in CI and GEDVI in responders (*P* < .05). There were no significant changes in MAP, CVP, EVLWI, or serum lactate concentration (not shown).

## Discussion

Our study represents the continuation of analyzing data from the previous investigation of fluid responsiveness^9^ and has shown the predictive role of SVV and PPV during sFCT in off-pump coronary surgery. Among a variety of invasive hemodynamic parameters, SVV and PPV have been accepted as reliable predictors of fluid responsiveness in different critical care scenarios including cardiosurgical patients.^21,22^ Notably, we observed an increase of CI after sFCT by >15% in less than half of patients after OPCAB that confirms the role of complex hemodynamic monitoring for guiding fluid therapy in this setting.^4^ Both PPV_PiCCO_ requiring femoral artery catheterization and less-invasive PPV_NK_ obtained from radial artery were able to predict the effects of standard fluid load. The decreased specificity of PPV_NK_ is probably related to differences in access for monitoring of arterial wave, leading to a more pronounced vasomotor increase in the radial artery with signal dampening.

Heart–lung interaction index has also shown a good predictive value for fluid responsiveness, an important benefit of this novel parameter is non-invasiveness. The HLI assessment is based on the amplitude of the plethysmogram variability that requires mechanical ventilation. The well-recognized limitations of this approach and other methods for monitoring of fluid responsiveness include spontaneous respiratory activity, open chest conditions, and low tidal volume.^23^ It is worth noting that we observed bradycardia below 50 beats/min as an additional limitation of HLI. Moreover, the conditions associated with decrease in peripheral perfusion such as shock, vasopressor support, hypothermia, and several other clinical situations, as well as motion artifacts can result in loss of pulse oximetry signal.^24^ These limitations are also a hallmark of PVI.^25^ In our study, we have not confirmed the usefulness of PVI in the detection of fluid responsive patients after OPCAB that is consistent with the results of Ganter et al.^15,16^ The absence of predictive role of PVI for fluid responsiveness in these studies can be related with specific characteristics of cardiosurgical patients including disorders of peripheral microcirculation, decreased cardiac output, and requirement in inotrope/vasopressor support.^15^ However, the study of Haas et al.^26^ demonstrated the satisfactory predictive ability of PVI after cardiopulmonary bypass. Notably, this study defined responders as the patients with increase in CI after fluid load by 10% but not by 15% like in other investigations.^15,16^ In addition, several studies confirmed the predictive value of PVI before surgery, but not postoperatively, when peripheral perfusion can be compromised by additional confounding factors.^25,27,28^ The discrepancy of our results regarding PVI in comparison with HLI is probably related to the difference in mathematical algorithms for calculation of these parameters.

There are some limitations of studied variables. In addition to perfusion abnormalities that can compromise the plethysmogram signal, one of them is the presence of arrhythmia, which frequently complicates the postoperative period of cardiac surgery and dramatically affects the accuracy of dynamic parameters. Thus, although atrial fibrillation was one of exclusion criteria, the interpretation of HLI for assessment of fluid responsiveness in our study was compromised by bradycardia observed in 22% of patients. 

In our study, the fluid responsiveness was not associated with main clinical outcomes. It is reasonable to argue that the augmentation in CI following fluid load is often transient and does not result in a steady increase of oxygen transport. Therefore, a positive value of dynamic parameter for fluid responsiveness should not automatically lead to fluid administration and must be assessed simultaneously with metabolic response and other hemodynamic characteristics.^23^

## Conclusions

In patients after off-pump coronary surgery, both invasive parameters such as pulse pressure and stroke volume variations and non-invasive parameter such as HLI are able to predict fluid responsiveness during the early postoperative period. Thus, these dynamic parameters can be used to guide fluid therapy during the early postoperative period of OPCAB.

## Figures and Tables

**Table 1.** Clinical Characteristics of the Patients

Characteristics	Responders, n = 14	Non-responders, n = 18	*P*
Age (years)	60 (53 to 72)	67 (58 to 74)	.12
EuroScore II (points)	1.1 (0.8 to 1.5)	1.4 (0.9 to 1.8)	.2
Ejection fraction before surgery (%)	58 (52 to 68)	60 (54 to 64)	.9
Anastomosis (number)	3 (2 to 4)	3 (2 to 3)	.09
Duration of surgery (min)	205 (184 to 240)	190 (170 to 212)	.16
Intraoperative fluid balance (mL)	600 (450 to 1118)	950 (550 to 1500)	.4
Fluid balance at 24 h after surgery (mL)	350 (–350 to 450)	–140 (–300 to 320)	.4
Duration of postoperative MV (min)	193 (143 to 264)	172 (115 to 238)	.3
Length of ICU stay (h)	48 (24 to 48)	24 (24 to 36)	.2

Data are presented as median (25th-75th percentile).

ICU, intensive care unit; MV, mechanical ventilation.

**Table 2.** The Patient Hemodynamics Before and After Standard Fluid Challenge Test

Characteristics	Group	Responders, n = 14	Non-responders, n = 18
SVV (%)	Before sFCT	12 (8-19)	6 (5-10)*
After sFCT	8 (4-13)^†^	6 (5-9)
PPV_PiCCO_ (%)	Before sFCT	13 (9-18)	6 (5-11)*
After sFCT	7 (4-11)^†^	6 (4-7)
PPV_NK_ (%)	Before sFCT	15 (10-21)	9 (7-15)*
After sFCT	9 (5-13)^†^	7 (6-15)
HLI (%)	Before sFCT	14 (5-21)	3 (2-8)*
After sFCT	7 (3-8)^†^	3 (2-6)
PVI (%)	Before sFCT	15 (10-21)	13 (11-18)
After sFCT	9 (5-13)^†^	11 (6-14)
GEDVI (mL m−2)	Before sFCT	610 (546-678)	717 (612-819)*
After sFCT	644 (561-807) ^†^	756 (606-921)
CI (mL min−1 m−2)	Before sFCT	1.9 (1.6-2.5)	2.3 (2.0-2.7)
After sFCT	2.5 (2.1-3.3)^†^	2.4 (2.0-2.9)

SVV, stroke volume variation; PPV_PiCCO_, pulse pressure variation assessed using femoral artery (PiCCO_2_); PPV_NK_, pulse pressure variation assessed using radial artery and Nihon Kohden patient monitor; HLI, heart–lung interaction index; PVI, plethysmogram variability index; sFCT, standard fluid challenge test; CI, cardiac index; GEDVI, global end-diastolic volume index.

^†^ *P *< .05 within the group, Wilcoxon test; *P < .05 between the groups, Mann–Whitney *U* test.
